# Composite Biomaterials Based on Sol-Gel Mesoporous Silicate Glasses: A Review

**DOI:** 10.3390/bioengineering4010015

**Published:** 2017-02-24

**Authors:** Francesco Baino, Sonia Fiorilli, Chiara Vitale-Brovarone

**Affiliations:** Department of Applied Science and Technology, Politecnico di Torino, Corso Duca degli Abruzzi 24, Torino 10129, Italy; sonia.fiorilli@polito.it (S.F.); chiara.vitale@polito.it (C.V.-B.)

**Keywords:** bioactive glass, mesoporous, composite, scaffold, tissue engineering

## Abstract

Bioactive glasses are able to bond to bone and stimulate the growth of new tissue while dissolving over time, which makes them ideal materials for regenerative medicine. The advent of mesoporous glasses, which are typically synthesized via sol-gel routes, allowed researchers to develop a broad and versatile class of novel biomaterials that combine superior bone regenerative potential (compared to traditional melt-derived glasses) with the ability of incorporating drugs and various biomolecules for targeted therapy in situ. Mesoporous glass particles can be directly embedded as a bioactive phase within a non-porous (e.g., microspheres), porous (3D scaffolds) or injectable matrix, or be processed to manufacture a surface coating on inorganic or organic (macro)porous substrates, thereby obtaining hierarchical structures with multiscale porosity. This review provides a picture of composite systems and coatings based on mesoporous glasses and highlights the challenges for the future, including the great potential of inorganic–organic hybrid sol-gel biomaterials.

## 1. Introduction

Bioactive glasses are amorphous materials that can bond to hard and soft tissues and stimulate the regeneration of healthy bone while dissolving over time, which makes them highly attractive candidates for healthcare and a 21^st^ century biomedicine [1]. The first bioactive glass, belonging to the 45SiO_2_–24.5Na_2_O–24.5CaO–6P_2_O_5_ (wt %) system (45S5 Bioglass^®^), was developed by Larry L. Hench at the University of Florida (USA) [2] and has been clinically used in a number of orthopedic and dental applications since the 1980s [3].

In the context of bone repair, “inorganic bioactivity” denotes the formation of a nano-crystalline apatite layer mimicking the mineral phase of bone on the implant surface, which is a precondition so that a tight bond to calcified tissues can occur [4]. While SiO_2_ should be less than 60 mol % in melt-derived glasses to allow the material to bond to bone as a consequence of the bioactive process [5], surface apatite formation was interestingly observed in glasses that contain up to 90 mol % of SiO_2_ if they are synthesized via a sol-gel process that creates an inherent mesoporosity (2–50 nm) in the material [6]. Hence, sol-gel glass particles are characterized by a high surface area available for the release of soluble ionic species (tens of m^2^/g compared to less than 1 m^2^/g of melt-derived glass products of equal size) and, thereby, bioactive reactions are significantly emphasized and accelerated [7].

**Figure 1:**
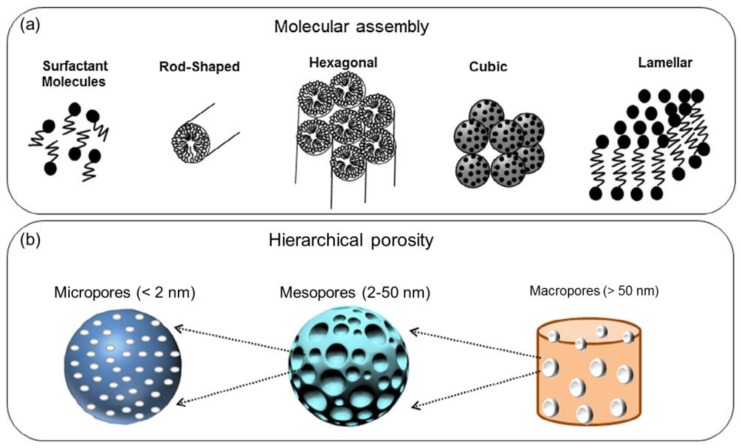
Basic concepts related to mesoporous materials: (**a**) molecular self-assembling of surfactant micelles to originate mesopores and (**b**) pore size scales according to IUPAC terminology. Images adapted from Owens et al. [15] © Elsevier.

Incorporation of supramolecular chemistry into the sol-gel process (Figure 1a) led to the advent of ordered silicate mesoporous materials [8], which have been proposed over the last 15 years as versatile platforms for the controlled release of drugs and growth factors that can be entrapped inside an ordered array of mesopores [9,10,11]. Classification of pore sizes according to International Union of Pure and Applied Chemistry (IUPAC) definitions [12] is schematically depicted in Figure 1b.

It is important to note that the so-called mesoporous bioactive glasses (MBGs) [13], which are typically synthesized by using a surfactant as mesopore template, are unique compared to “conventional” sol-gel bioactive glasses. In fact, although both types of glasses are produced by a wet method (sol-gel) and characterized by nanopores at the mesoscale (2–50 nm), the mechanism for the generation of the mesoporous texture is different: conventional sol-gel glasses possess mesopores which arise from the random distribution of CaO within the network of SiO_2_ [14]; on the contrary, the self-assembly of micelles in MBGs gives rise to uniform mesopores as well as ordered mesostructures with higher specific surface area (hundreds of m^2^/g). Under N_2_ adsorption–desorption analysis, conventional sol-gel glasses typically show H_2_-type hysteresis loops associated with inkbottle-like pores, while MBGs exhibit H_1_-type hysteresis loops typical of 1D spatially-ordered channels [13].

SiO_2_-based mesoporous materials, which are usually produced in the form of micro- or nano-sized particles, can be added to a polymeric or inorganic matrix by direct mixing or surface coating strategies in order to produce composites with suitable characteristics for medical applications, such as hierarchical 3D porous scaffolds or injectable cements. This review provides a state-of-the-art picture about the above-mentioned composites as well as a prospect for future research, highlighting the potential of hybrid systems in which the organic and inorganic phases are intimately linked at the nanoscale. In fact, hybrids allow many limitations of “traditional” nanocomposites to be overcome, keeping the best of the two components to produce a high-performance single-phase material with superior properties.

## 2. Hierarchical Glass-Based Composites Produced by “Mixing Strategies”

Incorporation of an inorganic phase into a polymeric matrix is a valuable strategy to improve the performance of the base material and impart extra-functionalities, primarily high strength and bioactivity. An overview of these applications is reported in Table 1.

**Figure 2:**
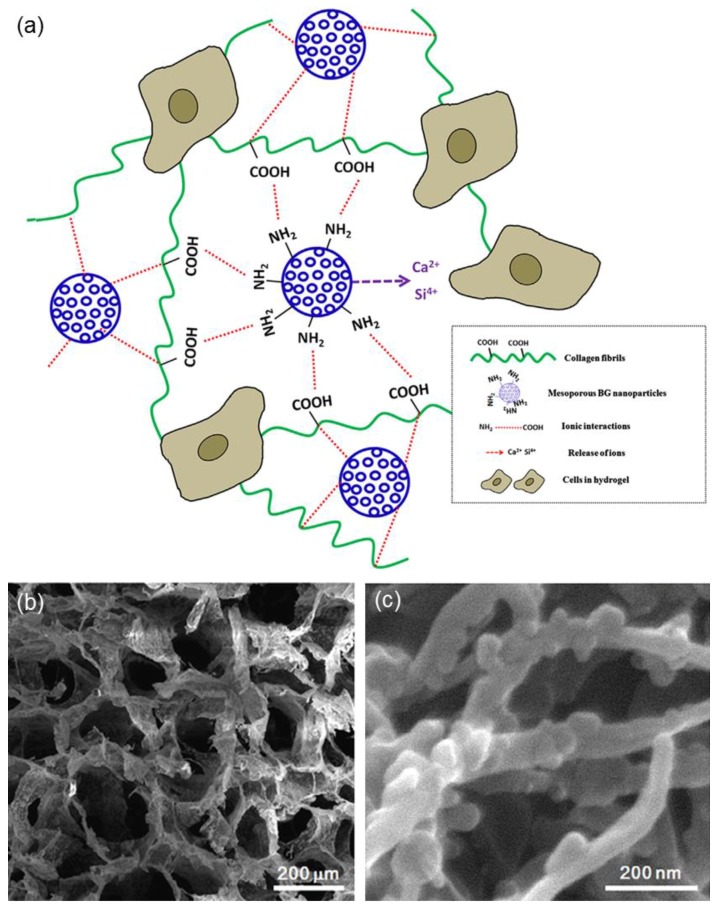
MBG/collagen nanocomposite obtained by freeze-frying: (**a**) schematic illustration of the material structure and function (ion release from MBG nanoparticles is expected to promote bioactivity and cellular mineralization, and the surface functionalization of MBG with amino groups facilitates chemical links with amino-acid sequences of collagen molecules, which enables the physico-chemical stability of the hydrogels); (**b**) scanning electron microscopy (SEM) micrograph of the hydrogel structure; and (**c**) detail showing the collagen fibrils integrated with MBG nanoparticles. Images adapted from El-Fiqi et al. [17] © Elsevier.

Collagen hydrogels, having similar composition to the bone extracellular matrix, are excellent platforms for the anchorage and spreading of cells, as well as for their subsequent proliferation and differentiation into target tissue lineages. However, one of the major drawbacks of collagen is its high rate of hydrolytic and enzymatic biodegradability, which limits the use of collagen hydrogels as biological constructs [16]. El-Fiqi et al. [17] showed that the addition of MBG nanoparticles (50–100 nm) as a second phase can be a valuable strategy to improve the performance of collagen hydrogels in terms of physico-chemical and mechanical properties (Figure 2) as well as biological behavior upon in vitro tests with mesenchymal stem cells.

Mesoporous silicate glass particles have been added as a bioactive phase to otherwise nearly-inert polymeric matrices in a number of studies. El-Kady et al. [18] incorporated sol-gel glass (60SiO_2_-36CaO-4P_2_O_5_ wt %) nanoparticles (size below 100 nm) into poly(L-lactic acid) (PLLA) scaffolds, produced by the solid–liquid phase separation method combined with solvent extraction, and observed the formation of a surface apatite layer upon in vitro tests; furthermore, the degradation rate of these composite scaffolds could be tailored according to the amount of silicate phase. Li et al. [19] prepared MBG/poly(lactic-*co*-glycolic acid) (PLGA) microspheres for obtaining a more sustained release of gentamicin over time. The same research group also experimented a solvent casting/particulate leaching route to fabricate MBG/poly(caprolactone) (PCL) composite scaffolds with improved drug release ability and high bioactivity in vitro [20]. For analogous purposes, MBG/alginate microspheres [21] and MBG/PLGA films [22] were prepared in other studies.

Su et al. used a solvent casting/particulate leaching strategy to produce MBG/polyamide scaffolds that exhibited excellent biocompatibility as well as superior bone regeneration potential in vivo (rabbit’s thighbone defects) compared to the polymeric material alone used as a reference [23].

**Figure 3:**
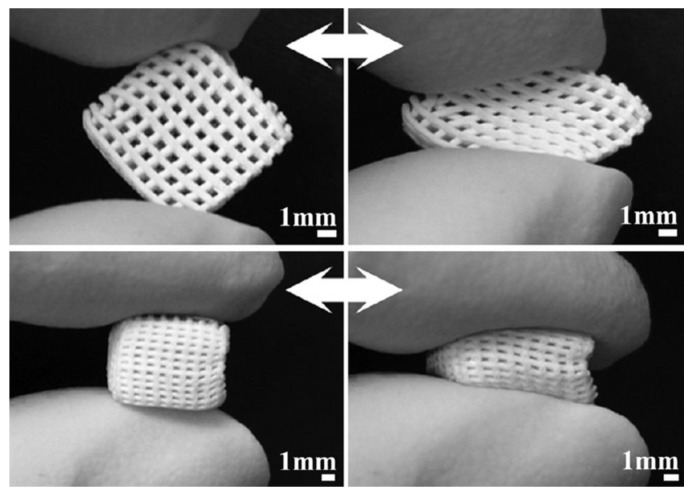
Compressibility of MBG/PCL composite scaffolds produced by robocasting (images adapted from Yun et al. [24] © Elsevier).

Yun et al. [24] combined salt leaching with robocasting to obtain MBG/PCL composite scaffolds exhibiting three-level multiscale porosity, i.e., giant macropores around 200 μm, mid-size macropores within 2 to 9 μm, and mesopores of 5 nm. The scaffolds were fabricated by directly extruding the gel paste onto a chilled substrate using a robotic deposition device. A gantry robocasting apparatus was used with three axes of motion control (x, y and z-axis) and a syringe was affixed as a reservoir on the z-axis motion stage. The gel paste housed in the syringe was deposited through a cylindrical nozzle (500 μm); a linear actuator served to depress the plunger of the syringe at a fixed speed so that the volumetric flow rate could be precisely controlled. The shapes and sizes of the scaffold as well as the arrangement of the macropores could be controlled by a computer system (Figure 3). These highly interesting constructs possessed adequate compressive strength (2–4 MPa) for bone repair, good in vitro bioactivity and a versatile sponge-like plastic nature with easy pliability, which suggested possible use as injectable scaffolds for minimally invasive surgery (Figure 3).

MBG/alginate pastes were also processed by 3D plotting [25] to fabricate hierarchical scaffolds provided with good biocompatibility, drug release properties and mineralization ability in vitro upon soaking in an inorganic simulated body fluid (SBF) that mimicked the composition of human plasma [4]. However, the mechanical properties were not completely satisfactory (1–1.5 MPa for as-such scaffolds and 0.5–0.8 MPa after immersion for 28 days in SBF) [25].

Silk fibroin and spray-dried MBG particles were also used to fabricate porous composites by freeze-drying: compared to the pure silk constructs, the hierarchical macro-mesoporous scaffolds exhibited an improved mechanical strength (up to 1 MPa) and a fast apatite-forming ability in vitro (hydroxyapatite agglomerates visible after 1 day in SBF) [26]. Similar results were obtained incorporating MBG particles obtained by a “conventional” wet synthesis into macroporous silk scaffolds [27].

Extending the application from organic/inorganic composites to biphasic ceramics, Li et al. [28] used a centrifugal embedding approach to fabricate a composite scaffold by combining MBG particles and calcium phosphate cement; bone morphogenetic proteins-2 (BMP-2) were also incorporated into this construct through a freeze-drying process. The obtained scaffolds exhibited a hierarchical pore structure (giant open pores of about 200 μm, macropores within 2 to 10 μm and mesopores of 5 nm), a nearly-adequate strength for bone repair (up to 1.4 MPa in compression), and supported the sustained release of BMP-2 over 7 days with excellent osteogenesis in a rabbit model.

As for the applications in soft tissue engineering, freeze-dried MBG/chitosan porous composite films were recently proposed as hemostatic membranes for wound healing [29]. Ultrathin MBG fibers (diameter of a few hundreds of nanometers) were prepared by Hong et al. [30] via electrospinning for possible use in skin tissue engineering; the same research group also demonstrated that the fibers could be prepared with a hollow core by a careful optimization of the processing parameters [31]. To the best of our knowledge, no other studies dealing with the fabrication of fibrous MBGs have been reported in the literature, perhaps due to the high cost associated to the electrospinning equipment needed for the mesoporous fiber production. Incorporation of such a bioactive phase in a polymeric matrix to obtain a MBG fiber-containing composite would deserve investigation in a future work.

## 3. Macroporous Scaffolds Provided with a Mesoporous Glass Coating

Deposition of a sol-gel glass coating on the struts of both inorganic and organic scaffolds was a key to impart extra-functionalities to the base 3D (macro)porous material—primarily bioactivity and drug delivery capability. An overview of these applications is reported in Table 2. 

Perhaps the first relevant study was reported by Miao [32] who dip-coated highly-porous (80 vol %) Al_2_O_3_ foams with a thin layer of “conventional” 58S sol-gel glass (60SiO_2_-36CaO-4P_2_O_5_ mol %) in order to impart apatite-forming ability to the otherwise nearly-inert ceramic skeleton. The same deposition strategy was carried out by Liu et al. [33] who applied a layer of 58S glass on the struts of 80Al_2_O_3_–20ZrO_2_ (vol %) ceramic scaffolds. The obtained hierarchical porous composite exhibited adequate porosity (60–66 vol % with large and interconnected macropores of 1–2 mm) and compressive strength (5–8 MPa) for bone substitution, along with good bioactive properties in vitro as revealed by the formation of a surface apatite layer after 1 day in SBF.

The presence of a sol-gel glass coating may also contribute to mechanically reinforce the structure of the base scaffold, as demonstrated by a few studies. A significant improvement of the mechanical properties of calcium phosphate scaffolds was achieved by applying a 58S/PCL composite coating (dipping procedure); specifically, it was observed that the compressive strength (0.2–1.5 vs. 0.1 MPa) and elastic modulus (20–50 MPa vs. 15 MPa) were strongly dependent on the amount of 58S glass (1–90 wt %) introduced into the coating [34]. In another study, Esfahani et al. [35] coated highly-porous (83 vol %) scaffolds of bovine-derived hydroxyapatite with a thin layer of 58S glass by a prolonged dipping procedure to increase the compressive strength of the material from 0.22 to 1.49 MPa.

**Figure 4:**
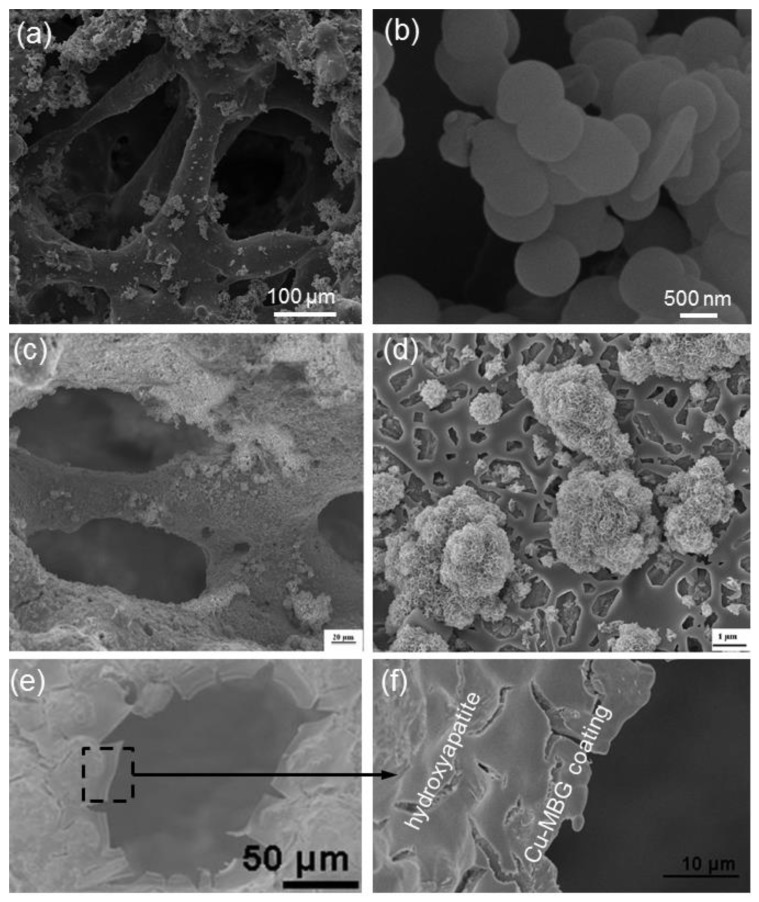
Ceramic scaffolds provided with a mesoporous coating (SEM micrographs are reported): (**a**) MCM-41 spheres deposited on a bioactive glass–ceramic foam; and (**b**) detail of the pure silica mesoporous particles anchored on the scaffold struts (images adapted from Vitale-Brovarone et al. [38] © Springer); (**c**) CaSiO_3_-based scaffold coated with a MBG coating deposited by electrophoretic deposition to improve bioactivity, as demonstrated by the surface apatite agglomerates formed after 2 days in SBF (simulated body fluid) (**d**) (images adapted from Fiorilli et al. [45] © Springer); (**e,f**) Cu-doped MBG layer applied on a hydroxyapatite orbital implant to impart antibacterial properties (images adapted from Ye et al. [49] © Springer)..

Besides the “conventional” sol-gel glasses, mesoporous materials (both pure amorphous SiO_2_ and MBGs), too, were deposited as smart coatings on the struts of a variety of 3D scaffolds with the specific purpose of obtaining multifunctional systems that could combine bioactivity, mechanical support to the host and regenerated bone tissue, and drug release properties. The first prototype of such a construct comprised a bioactive glass–ceramic macroporous foam coated with a micrometric layer of SBA-15 spheres (mesopores within 5 to 8 nm) [36]. This early study was then extended to MCM-41 (Figure 4a,b) that, having a narrow size of mesopores (2–3 nm), should have allowed a finer control of the release kinetics of ibuprofen (drug molecule size about 1 nm) [37,38,39]. In both cases, the pure SiO_2_ mesoporous glass coating played a key role, on the one hand in enhancing the drug adsorption ability of the porous construct and, on the other hand, in allowing a long-term release of the therapeutic molecules. A similar approach was also proposed by Boccardi et al. [40] who synthesized submicronic MCM-41 spheres directly on 45S5 Bioglass^®^-derived glass–ceramic scaffolds through a modified Stöber method.

From the viewpoint of regenerative medicine, the major limitation of pure SiO_2_ mesoporous materials is their moderate reactivity in contact with biological fluids: in fact, although MCM-41 and SBA-15 were shown able to form a surface apatite layer upon prolonged immersion in SBF [41,42], the reaction kinetics are too slow to consider them as effective materials for bone repair. With these concerns in mind, researchers introduced other oxides (primarily CaO and P_2_O_5_) into the silica network to obtain MBGs with exceptional apatite forming ability [43]. These materials have been often proposed in the form of fine particulate or somehow processed (e.g., sponge replication, 3D printing) to obtain 3D macro-mesoporous scaffolds [44], and more rarely have been applied as coatings on preexisting porous substrates. Recently, MBG particles were deposited on CaSiO_3_-containing macroporous foams by the electrophoretic mechanism in order to combine the excellent bioactivity of the mesoporous phase (Figure 4c,d) with the high mechanical strength (above 15 MPa under compressive loads) of the glass–ceramic skeleton [45]. A similar approach was also employed to deposited spray-dried Sr-containing MBG spheres on glass–ceramic scaffolds to improve bone regeneration as strontium is known to exhibit osteogenic and bone antiresorptive properties [46].

With the same purpose in mind, Zhang et al. [47] used a spin coating method to deposit a thin layer (about 100 nm) of nano-sized MBG on the struts of a 3D-printed β-tricalcium phosphate (TCP) scaffold to improve its bone regenerative potential, which was actually confirmed by in vivo tests in rabbits.

Shi et al. [48] fabricated MBG/PLGA-coated CaSiO_3_ scaffolds by dipping the macroporous template in a polymer solution with different amounts of suspended nano-sized MBG particles. Compared to PLGA/CaSiO_3_ scaffolds, the compressive strength significantly increased due to the presence of MBG particles embedded in the surface coating (1.5–2 vs. 0.4 MPa). Furthermore, the bioactive surface layer enhanced the in vitro mineralization ability, the proliferation and early cell differentiation of osteoblasts, and allowed a controlled release of ibuprofen to be achieved.

In a very interesting study, Ye et al. [49] coated hydroxyapatite porous orbital implants with a Cu-doped MBG coating to combine the bactericidal effects of Cu^2+^ ions released as the coating degrades and ofloxacin, an antibiotic which was encapsulated in the mesopores of the glass (Figure 4e,f). This composite device exhibited antibacterial activity against both gram-positive (*Staphylococcus aureus*) and gram-negative bacteria (*Escherichia coli*), thereby opening new perspectives for the prevention and treatment of implant-related ocular infections.

In the field of bioresorbable scaffolds based on phosphate glasses, Novajra et al. [50] recently developed fibrous scaffolds obtained by thermally bonding non-porous phosphate glass fibers enriched with MBG particles. The developed scaffolds showed a compressive strength of 3.5 MPa, a very high bioactivity and drug release ability due to the presence of mesoporous particles on the fiber surface.

While abundant literature can be found about the development of glass/polymer porous composites, especially when comprising the well-known (non-porous) 45S5 Bioglass^®^ [51,52], there is a paucity of studies dealing with polymeric scaffolds coated by mesoporous glass. Zhu et al. [53] applied a layer of MBG particles on the surface of macroporous poly(L-lactic acid) (PLLA) scaffolds by dip-coating in order to impart bioactive properties and drug uptake/release capability to the polymeric skeleton. For the same purpose, MBG particles were also deposited on the struts of poly(3-hydroxybutyrate-*co*-3-hydroxyhexanoate) scaffolds obtained by 3D printing [54].

An interesting application, which can be actually considered an optimization of the sponge replica method to produce cellular glasses, was recently reported by Cabanas-Polo et al. [55] who exploited the electrophoretic mechanism in the processing of 60.8SiO_2_-30.9CaO-5.8Na_2_O-2.4P_2_O_5_ (mol %) sol-gel glass foams. In this electrophoresis-guided approach, the sol precursor molecules (positively charged) travelled from the surroundings of the counter-electrode to a Ni wire fixed to a polyurethane foam (cathode), thereby allowing a homogeneous impregnation of the porous substrate to be obtained. At the end of the process, the sol-coated polymeric template was left to dry and thermally-treated to obtain a sintered glass replica of the foam.

Occasionally, sol-gel glass has also been used to produce coatings on the surface of metallic porous substrates. From a general viewpoint, fabrication of bioactive glass monolithic coatings on metal implants is challenging due to a series of problems associated with high-temperature sintering required for the consolidation of the bioactive layer, including the risk of oxidation of the metal surface and the mismatch between the thermal expansion coefficients of metal and glass. A solution to these issues comes from the development of polymer/glass coatings that can adhere to the metallic substrate at low temperatures. An interesting study was reported by Yazdimamaghani et al. [56] who applied a PCL/gelatin coating reinforced with 64SiO_2_-31CaO-5P_2_O_5_ (mol %) sol-gel glass particles on the surface of magnesium scaffolds by means of a freeze-drying process. The presence of the bioactive coating induced the growth of a surface apatite layer on the scaffold struts and, perhaps most importantly, played a protective role on the metal lying underneath. In fact, it was observed that pure magnesium scaffolds were fully degraded after 3 days in SBF, whereas about 87 wt % of PCL/gelatin/glass-coated samples remained after immersion for 14 days in the testing solution.

## 4. Sol-Gel Glass Coatings on Prosthetic Devices

Bioactive glass coatings have been usually produced by enameling on the surface of metallic and ceramic prostheses [57]; however, this approach is not very adequate if sol-gel glasses have to be processed as the high-temperature firing step required for the sintering and consolidation of the coating could involve the elimination of the nanoporosity. Perhaps this was the reason why—along with some general concerns about the long-term adhesion of bioactive glass coatings to metallic prostheses [58]—the research involving sol-gel glasses was primarily addressed to other applications (e.g., 3D scaffolds), except for a few studies. Schrooten and Helsen [59] combined an experimental approach with SEM analysis to evaluate the adhesion strength of plasma-sprayed bioactive 52SiO_2_-30.5CaO-9.8Na_2_O-6.2P_2_O_5_-1.5CaF_2_ (mol %) sol-gel glass coatings on titanium dental implants. The coating was able to withstand a maximum tensile stress of 47 MPa; adhesion tests performed after 2 months in SBF revealed that the adhesion strength of the coating decreased by about 10% but the interface between the glass and titanium implant remained fully intact at all times. However, no evidence of the persistence of the mesoporous structure in the glass after firing was reported.

Esfahani et al. [60] dip-coated nickel-titanium (Nitinol^®^) nails with a composite layer made of nano-sized 58S glass and titanium oxide (TiO_2_) produced via a sol-gel route. The 58S particles (50–60 nm) dispersed in the TiO_2_ matrix enhanced the hardness of the coating and a direct pull-out test recorded a coating–substrate bonding strength larger than 16 MPa. Furthermore, in vitro bioactivity studies showed the formation and growth of hydroxyapatite agglomerates on the surface of the 58S/TiO_2_-coated Nitinol^®^ alloy upon soaking in SBF, while the as-such implant had a bioinert behavior.

## 5. Injectable Cements Containing Mesoporous Silicate Materials

Implant fixation to the patient’s host bone is vital to ensure the long-term success of an orthopedic joint prosthesis. At present, the most popular strategy involves the use of acrylic bone cement that acts as a grouting material at the bone–implant interface [61]. A variety of materials, such as carbon nanotubes [62] and TiO_2_ fibers [63], have been incorporated within the powder or monomer component prior to mixing in the attempt to enhance the static and dynamic mechanical properties of the final bone cement. Despite promising in vitro results [64], however, issues concerning the interfacial adhesion between acrylic matric and reinforcing phase, high stiffness and poor handling characteristics have generally prevented these composite cements from transitioning from the bench to clinical applications [65].

**Figure 5:**
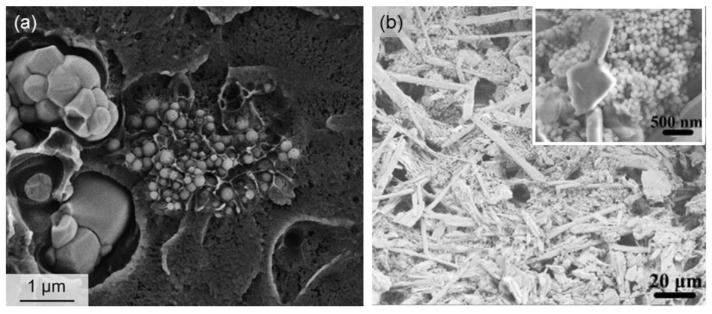
SEM micrographs of mesoporous silica nano-spheres incorporated in bone cements with a matrix of (**a**) poly(methyl methacrylate) (image adapted from Slane et al. [67] © Elsevier) or (**b**) calcium sulfate needles (image adapted from Li et al. [68] © Elsevier).

In the last few years, mesoporous materials have also been proposed as a second phase to add to bone cement for overcoming the above-mentioned challenges. Slane et al. [66] incorporated different amounts of mesoporous silica nanoparticles (MSNs) (0.5, 2 and 5 wt %) to a commercially-available acrylic cement and evaluated the impact on the static mechanical properties, fatigue life and hydration properties. It was reported that the flexural and compressive moduli, the compressive strength and the hydration degree increased with increasing concentration of MSNs, whereas the flexural strength and fracture toughness significantly decreased. The obtained results suggested that the interfacial adhesion strength between MSNs and acrylic matrix was poor, thereby leading to a decrease in the flexural and fatigue properties; problems of mixing between the two phases were also reported. The dispersion of the particles within the polymer was improved by the surface functionalization of the MSNs with amino groups (Figure 5a) [67].

In another study, Li et al. [68] prepared composite cement pellets by mixing calcium sulfate α-hemihydrate and MSNs, which were surface-functionalized with aminopropyltriethoxysilane (APS) to significantly improve the uptake and release of vancomycin (Figure 5b). These composite pellets showed no pyrogenic effect, met the clinical requirements on hemolytic reaction and were highly biocompatible as demonstrated by early in vitro biological tests.

A very interesting application of mesoporous materials in the development of innovative bone cements was recently proposed by Tallia et al. [69], who synthesized radiopaque and bioactive particles of ZrO_2_-containing MBG that can be added to injectable materials for minimally invasive surgery procedures (e.g., acrylic cement or calcium sulfate), in order to allow the visualization of the implant under fluoroscopic control during both injection and follow-up. This research activity, based on a patent deposited by Vitale-Brovarone et al. [70], has led to the development of an injectable bioresorbable and bioactive cement (Spine-Ghost). Specifically, Spine-Ghost is a composite cement formed by a resorbable calcium sulfate matrix enriched by spray-dried MBG particles and a radiopaque glass–ceramic phase. Spine-Ghost showed excellent mechanical strength, a very high bioactivity, adequate setting times for use in vertebral surgery and radiopacity comparable to the commercial reference Cerament^®^ [71]. It was successfully tested on an ad hoc developed sheep vertebral model showing suitable cement resorption and excellent bone formation [72].

## 6. Other Types of Composites and Coatings

Given the high brittleness of sol-gel scaffolds due to their inherent nanoporosity, deposition of a silk coating on the struts of MBG foams was rightly thought to improve the mechanical strength of the material, although this occurred to a still insufficient extent for safe handling and implantation (from 60 to 250 kPa, which is one order of magnitude below the strength of cancellous bone) [73]. Another application of polymer-coated MBG foams was reported by Ma et al. [74] who deposited a PLGA-magnetic SBA-15 coating on the struts of a cattail-templated MBG scaffold for the dual release of ibuprofen and metformin.

**Figure 6:**
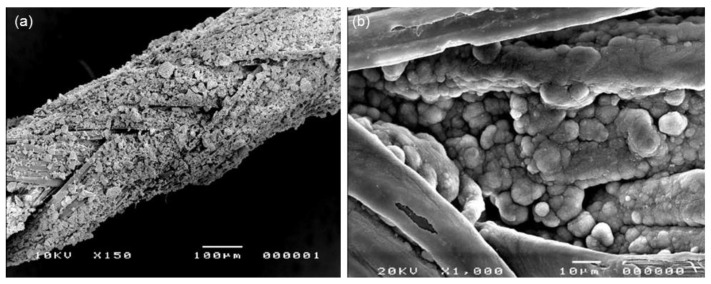
SEM micrographs of a Mersilk^®^ suture coated with Ag-doped sol-gel bioactive glass powders (**a**) before and (**b**) after soaking for 7 days in SBF (hydroxyapatite agglomerates are clearly visible on the suture surface). Images adapted from Blaker et al. [75] © Elsevier.

Sol-gel glasses have also found application in soft tissue repair in the form of bioactive coatings applied on polymeric sutures for wound dressing. Blaker et al. [75] coated Vycril^®^ and Mersilk^®^ resorbable sutures with Ag-doped sol-gel glass powder (60SiO_2_–34CaO–4P_2_O_5_–2Ag_2_O mol %) by a dipping procedure and reported the good in vitro bioactive behavior and bactericidal effect of the composite (Figure 6). In another study on the same materials, Pratten et al. [76] compared the antimicrobial activity elicited by Mersilk^®^ sutures coated with the same Ag-doped glass with that of 45S5 Bioglass^®^-coated sutures against *Staphylococcus epidermidis*, and reported that Ag-doped samples exhibited a significantly greater effect in limiting bacterial attachment.

## 7. Beyond Multiphasic Composite Materials: Hybrid Systems

Over the last decade, there has been much interest to go beyond the classical concept of composite and nanocomposite materials, the components of which are distinguishable by a clear interface and each of which maintain their peculiar physico-chemical characteristics. The challenge has been the development of inorganic–organic hybrid materials in which the components interact at the molecular level and are indistinguishable above the nanoscale [77]. This intimate interaction should allow cells to come into contact with both phases at one time, and the material should degrade at a single rate without having mismatched resorption kinetics of inorganic and organic phases [78].

Hybrids are typically synthesized by introducing a polymer in the sol-gel process after hydrolysis of the silica precursor alkoxide (e.g., tetraethyl orthosilicate (TEOS)), so that the inorganic silica network can form around the polymer molecules and nanoscale interactions between the two components can be created. Compared to sol-gel glasses, ageing and drying must be performed to a significantly lower temperature (below 100 °C) to avoid degradation of the system. In general, bioactive sol-gel hybrids are expected to have bioactivity similar to that of bioactive glasses but superior toughness and controlled congruent degradation [79]. This new class of biomaterials has been recently reviewed in detail by Jones [80] and Owens et al. [15].

Hybrid sol-gel materials can be classified into two classes depending on the interactions between the inorganic and organic chains [76]. Class I comprises hybrids that do not contain covalent or ionic-covalent bonds between the organic and inorganic phases, but each component interacts by only weak interactions, such as H-bond, Van der Waals bond, π–π interaction or electrostatic forces. Typically, the polymer is “mechanically” entrapped in the silica network during condensation, and the inorganic and organic chains are held together by H-bonding to the Si–OH surface groups. Biocompatible and degradable polymers of natural and synthetic origin have been used for biomedical applications, such as poly(vinyl alcohol) (PVA) [81,82], PCL [83,84], gelatin [85] and chitosan [86,87,88].

**Figure 7:**
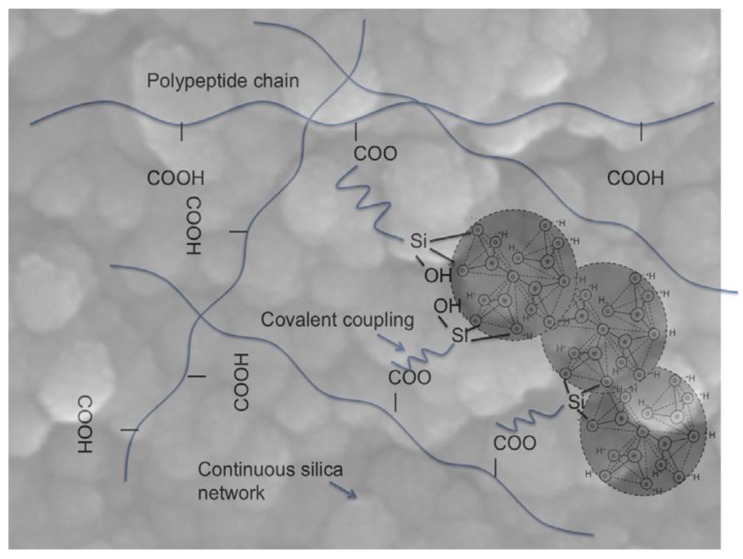
Schematic illustration of the interpenetrating inorganic and organic networks of a typical class-II hybrid material. Three nanoparticles of the continuous silica network are highlighted. The polymer chains are bonded to the silica network by a coupling agent (3-glycidyloxypropyl)trimethoxysilane (GPTMS)). Carboxylic acid groups on the polymer act as nucleophiles to open the epoxy ring of the GPTMS and form a bond. Image adapted from Jones [79] © Elsevier.

On the contrary, class-II hybrids include materials in which a part of the organic and inorganic components is linked to each other by strong chemical bonds such as covalent or ionic-covalent bonds (Figure 7). For this purpose, we can use either a polymer that already contains silane bonds (e.g., poly(dimethoxysilane) [89]) or the polymer can be functionalized with a coupling agent before being introduced in the sol-gel process (e.g., poly(ethylene glycol) (PEG)-SiO_2_ class-II hybrids synthesized by using triethoxysilyl-terminated PEG and TEOS [90]).

The synthesis of an interesting type of hybrids, called “cerasomes”, was recently reported by Katagiri et al. [91]; these nanostructures mimic natural liposomes and were produced by a dual process of sol-gel and self-assembly of organoalkoxysilanes. Potential applications include the modelling of biological membranes to investigate the diffusion of ions and lipophilic substances through them [92] as well as the development of new drug delivery systems since cerasomes are able to incorporate different organophylic molecules [93].

A very special type of class-II hybrids includes the so-called “star gels”, which have an organic core surrounded by flexible arms terminated in alkoxysilane groups [94,95] that form a silica-like network via hydrolysis and polycondensation. These materials typically hold the excellent bioactivity and biocompatibility of sol-gel glasses but exhibit a significantly superior toughness, thereby showing promise towards the development of bone-like self-repairing biomaterials [96].

The development of hybrid biomaterials also has the potential to overcome the issues related to the toxicity of (inorganic) nanomaterials. In fact, concerns exist about the fate of nano-sized materials in vivo, including the mesoporous silica and bioactive glass nanoparticles released from composite materials while the polymeric matrix progressively degrades [97]. At present, a clear consensus in the scientific community about this topic does not exist yet and some studies report controversial or partial results [98]. Hudson et al. [99] investigated the toxicity of MCM-41 and MCM-48 in rats, and reported, on the one hand, a good biocompatibility if the silica particles were injected subcutaneously at the sciatic nerve but, on the other hand, a lethal effect after intra-peritoneal or intra-venous injection, which suggested the risk of systemic disease associated to submicronic silica. Guidi et al. [100] analyzed the biological effect of MCM-41 nanoparticles (250 and 500 nm) in vitro in the presence of A549 and RAW264.7 cell types. It was observed that MCM-41 spheres were incorporated within endocytic vacuoles and induced DNA strand breaks and chromosomal alterations in both cell lines.

In summary, implantation of hybrid materials that can behave as a single phase and, thus, undergo congruent dissolution generating ionic products or residues which can be safely metabolized by living tissues and organs could eliminate the risk of the suspected genotoxic effect related to the size and dosage of silicate nanomaterials.

## 8. Conclusions

The advent of MBGs revolutionized tissue engineering as these biomaterials allow the combination of bone bonding and tissue regeneration abilities with the incorporation (and subsequent release) of drugs and growth factors inside the mesopores. If the presence of an ordered mesoporosity is indeed the strength of MBGs—being responsible for the excellent bioactivity in vitro and in vivo and providing the means to incorporate therapeutic biomolecules—on the other hand, it also represents the major weakness of these materials, since it implies a certain sacrifice of the mechanical properties. This drawback can be mitigated if MBGs are not used alone but are incorporated, for example, in a polymeric matrix to produce a composite or are applied as a coating on a stronger substrate, such as a glass–ceramic scaffold. However, MBG-containing composites still suffer from some limitations, including interfacial weakness between the components, different degradation rates between the organic and inorganic phase, and risk of toxicity associated to the nano-sized mesoporous products released into the body. The latest research directions are addressed to overcoming these problems by the development of inorganic–organic hybrid systems, which degrade at a single rate, are tough and hold the high bioactivity of sol-gel glasses.

## 9. Methods of Literature Search

A search of the SCOPUS database was conducted from 2000 to 2016 with a subsequent review of accompanying references. Major keywords used singly or in combination included composite, sol-gel, bioactive glass, mesoporous, hierarchical, silicate, scaffold, coating, cement, tissue engineering, and hybrid. Additional references were taken from the bibliography of the references identified through the SCOPUS search, and only the most pertinent have been referenced here.

## Figures and Tables

**Table 1:** Hierarchical composites containing a mesoporous glass phase.

Mesoporous Phase	Matrix	Processing	Effect	References
MBG	Collagen	Mixing	Improved bioactivity in vitro and mechanical properties	[17]
Sol-gel glass	PLLA	Solid–liquid phase separation + solvent extraction	Improved bioactivity in vitro	[18]
MBG	PLGA	Mixing	Improved bioactivity in vitro, drug release	[19,22]
MBG	Polyamide	Solvent casting/particulate leaching	Improved bioactivity in vitro and bone regeneration in vivo	[23]
MBG	PCL	Solvent casting/particulate leaching	Improved bioactivity in vitro, drug release	[20]
		Robocasting + salt leaching	Improved bioactivity in vitro and mechanical properties, pliability	[24]
MBG	Alginate	Mixing	Improved bioactivity in vitro, drug release	[21]
		3D plotting	Improved bioactivity in vitro, drug release	[25]
MBG	Silk fibroin	Freeze-drying	Improved bioactivity in vitro and mechanical properties	[26,27]
MBG	Calcium phosphate	Mixing + freeze-drying	Adequate mechanical properties and bone regeneration in vivo	[28]
MBG	Chitosan	Freeze-drying	Hemostasis to promote wound healing	[29]

MBG: mesoporous bioactive glasses; PLLA: poly(L-lactic acid); PLGA: poly(lactic-co-glycolic acid); PCL: poly(caprolactone).

**Table 2:** Scaffolds provided with a mesoporous coating.

Coating	Base scaffold	Processing	Effect	References
58S	Al_2_O_3_	Dip-coating	Improved bioactivity in vitro	[32]
58S	Al_2_O_3_/ZrO_2_	Dip-coating	Improved bioactivity in vitro	[33]
58S/PCL	Calcium phosphate	Dipping	Improved mechanical properties	[34]
58S	Bovine hydroxyapatite	Dipping	Improved mechanical properties	[35]
SBA-15	Glass–ceramic	Dipping	Drug release	[36]
MCM-41	Glass–ceramic	Dipping	Drug release	[37,38,39]
MBG	Glass–ceramic	Electrophoretic deposition	Improved bioactivity in vitro	[45,46]
MBG	β-TCP	Spin coating	Improved bioactivity in vitro	[47]
MBG/PLGA	CaSiO_3_	Dipping	Improved bioactivity in vitro and mechanical properties, drug release	[48]
Cu-doped MBG	Hydroxyapatite	Dipping	Drug release and antibacterial effect due to the release of Cu^2+^ ions	[49]
MBG	Phosphate glass fibers	Thermal bonding	Improved bioactivity in vitro, drug release	[50]
MBG	PLLA	Dip-coating	Improved bioactivity in vitro, drug release	[53]
PCL/gelatin/sol-gel glass	Magnesium	Freeze-drying	Improved bioactivity in vitro, reduction of magnesium dissolution	[56]
